# Peroxisome proliferator-activated receptor γ coactivator 1α regulates downstream of tyrosine kinase-7 (Dok-7) expression important for neuromuscular junction formation

**DOI:** 10.1038/s41598-024-52198-x

**Published:** 2024-01-20

**Authors:** Takumi Sugimoto, Chihiro Sakamaki, Tokushi Kimura, Takahiro Eguchi, Shinji Miura, Yasutomi Kamei

**Affiliations:** 1 Laboratory of Molecular Nutrition, Graduate School of Environmental and Life Science, Kyoto Prefectural University, Kyoto, Japan; 2 Brain-Skeletal Muscle Connection in Aging Project Team, Geroscience Research Center, National Center for Geriatrics and Gerontology, Aichi, Japan; 3 Laboratory of Nutritional Biochemistry, Graduate School of Nutritional and Environmental Sciences, University of Shizuoka, Shizuoka, Japan

**Keywords:** Neurology, Endocrine system and metabolic diseases, Metabolism, Neurophysiology

## Abstract

The neuromuscular junction (NMJ)—formed between a motor nerve terminal and skeletal muscle fiber—plays an important role in muscle contraction and other muscle functions. Aging and neurodegeneration worsen NMJ formation and impair muscle function. Downstream of tyrosine kinase-7 (Dok-7), expressed in skeletal muscle fibers, is essential for the formation of NMJ. Exercise increases the expression of the transcriptional coactivator peroxisome proliferator-activated receptor γ coactivator 1α (PGC1α) in skeletal muscles and restores NMJ formation. In this study, we used skeletal muscle-specific PGC1α knockout or overexpression mice to examine the role of PGC1α in regulating Dok-7 expression and NMJ formation. Our findings revealed that Dok-7 expression is regulated by PGC1α, and luciferase activity of the Dok-7 promoter is greatly increased by coexpressing PGC1α and estrogen receptor-related receptor α. Thus, we suggest PGC1α is involved in exercise-mediated restoration of NMJ formation.

## Introduction

The neuromuscular junction (NMJ) is formed between a motor nerve terminal and skeletal muscle fiber and is important for muscle functions, such as muscle contraction. Aging and neuromuscular diseases such as amyotrophic lateral sclerosis and duchenne muscular dystrophy, have been reported to cause neurodegeneration and acetylcholine receptor (AChR) fragmentation^1–3^. Degradation of NMJ structure precedes the onset of age-related loss of muscle mass and strength (sarcopenia), leading to impaired muscle function^4^. Therefore, the NMJ structure must be preserved to maintain quality of life and extend a healthy lifespan. Downstream of tyrosine kinase-7 (Dok-7) is expressed on the skeletal muscle side of the NMJ. Dok-7 promotes AChR clustering by facilitating phosphorylation of the downstream muscle-specific kinase^5,6^. Dok-7 overexpressing mice showed enhanced NMJ formation, and NMJ formation was impaired in Dok-7 knockout mice^5,7^. Exogenous Dok-7 expression suppressed motor nerve terminal degeneration and loss of exercise capacity due to abnormal NMJ formation seen mouse models of aging and amyotrophic lateral sclerosis^8,9^. Thus, Dok-7 is essential for NMJ formation.

The expression of the transcriptional coactivator peroxisome proliferator-activated receptor γ coactivator 1α (PGC1α) increases in the skeletal muscle during exercise^10^. Several signaling pathways, including Ca^2+^-dependent signaling, ROS, NO, AMPK, and p38 MAPK, have been implicated in the regulation of PGC1α expression and function in skeletal muscle. Recent findings suggest that p38 MAPK signaling is functionally required for endurance exercise-induced PGC1α regulation^11,12^. Recently, a clock gene has been suggested to be involved in the exercise-induced increase in PGC1α expression^13^. PGC1α promotes a variety of exercise-related metabolic processes, including mitochondrial biogenesis and fatty acid oxidation^10,14–16^. PGC1α regulates the expression of target genes by activating nuclear receptors such as estrogen receptor-related receptors (ERRs)^17^. Our comprehensive gene expression analysis of skeletal muscle-specific PGC1α knockout (PGC1α-mKO) mice showed decreased expression of Dok-7^18^. The expression of genes involved in NMJ formation was reported to increase in PGC1α-overexpressing cells and mice. Increased PGC1α expression ameliorated muscle damage in mouse models of duchenne muscular dystrophy^19,20^. However, the relationship between PGC1α and Dok-7 is unknown.

In this study, we investigated the role of PGC1α and ERRα in regulating Dok-7 expression and NMJ formation.

## Results and discussion

### Skeletal muscle-specific PGC1α knockout decreased Dok-7 expression in mice

**Figure 1:**
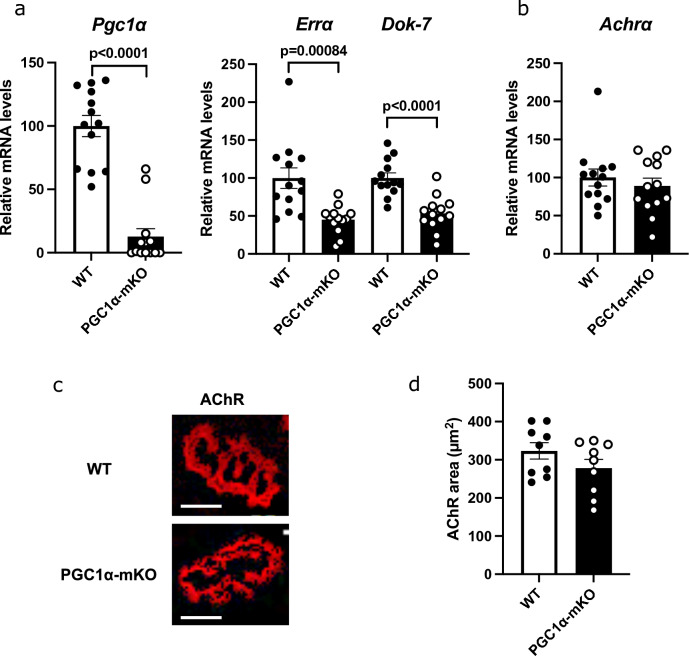
Reduced downstream of tyrosine kinase-7 (Dok-7) gene expression in skeletal muscle-specific peroxisome proliferator-activated receptor γ coactivator 1α knockout (PGC1α-mKO) mice. (**a**, **b**) Gene expression analysis of the gastrocnemius muscle from 8-week-old male mice and 22- to 29-month-old female mice using quantitative real-time PCR (N = 13). Data were normalized to 36B4 expression and expressed relative to wild-type (WT) mice. (**c**) AChR staining of the extensor digitorum longus muscle. Representative images are shown in each group (N = 9). Scale bar = 15 µm. (**d**) The size (area) of AChR clusters was quantified (N = 9). The values are shown as the mean ± SE. The coefficients of variation are shown in Supplementary Table 2.

Gene expression was analyzed in the gastrocnemius muscle of PGC1α-mKO mice. The expression of PGC1α, ERRα, and Dok-7 were all decreased in PGC1α-mKO mice (Fig. 1a), while AChRα gene expression and AChR area (at the neuromuscular junction) were unchanged (Fig. 1b–d). By contrast, others have reported reduced AChRα gene expression in the gastrocnemius muscle of PGC1α-mKO mice^19^. We also performed gene expression analysis on the extensor digitorum longus, plantaris, soleus and tibialis anterior muscles, and NMJ staining on the extensor digitorum longus muscle of PGC1α-mKO female mice (Supplementary Fig. 1). The gene expression obtained in several types of muscles was similar to that obtained in Fig. 1. The mean AChR area of PGC1α-mKO female mice was unchanged (Supplementary Fig. 1e,f).

### Skeletal muscle-specific PGC1α overexpression increased Dok-7 expression and enhanced NMJ formation in mice

**Figure 2:**
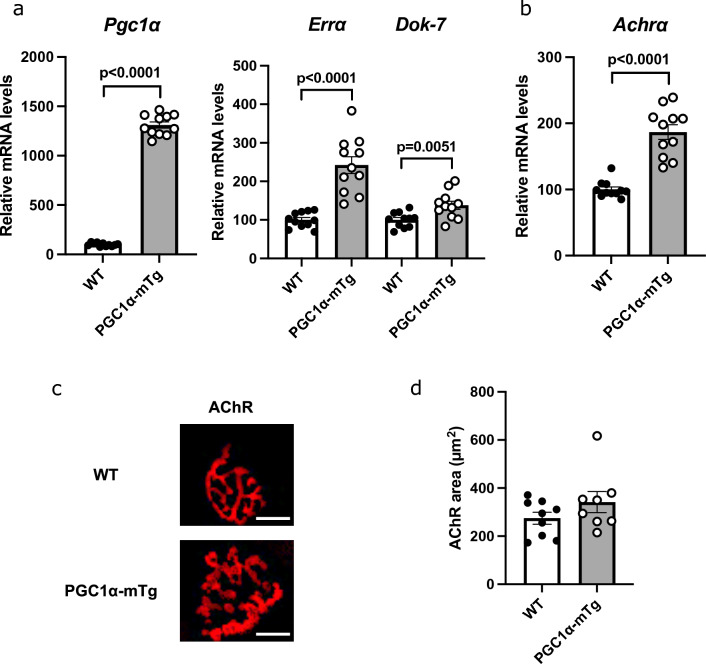
Increased downstream of tyrosine kinase-7 (Dok-7) gene expression in skeletal muscle-specific peroxisome proliferator-activated receptor γ coactivator 1α (PGC1α) overexpression (PGC1α-mTg) mice. (**a**, **b**) Gene expression in the gastrocnemius muscle of 10–12-week-old male mice and 8-week-old female mice was analyzed using quantitative real-time PCR (N = 11). Data were normalized to 36B4 expression and expressed relative to wild-type (WT) mice. (**c**) AChR staining of the extensor digitorum longus muscle. Representative images are shown in each group (N = 8 or 9). Scale bar = 15 µm. (**d**) The area of AChR clusters was quantified (N = 8 or 9). Values are shown as the mean ± SE. The coefficients of variation are shown in Supplementary Table 2.

Gene expression was analyzed in the gastrocnemius muscle of skeletal muscle-specific PGC1α overexpression (PGC1α-mTg) mice. Gene expression of PGC1α, ERRα, and Dok-7 (Fig. 2a), as well as AChRα (Fig. 2b), increased in PGC1α-mTg mice. The mean AChR area was larger in PGC1α-mTg mice, although the difference was not significant (*p* = 0.19) (Fig. 2c,d). Because NMJs form between motor nerve terminal and skeletal muscle fibers, the nerves of PGC1α-mTg mice were also stained. The location of AChR and nerve coincided in both PGC1α-mTg and wild-type (WT) mice, indicating normal NMJ formation (Supplementary Fig. 2). We also performed gene expression analysis on the extensor digitorum longus, plantaris, soleus and tibialis anterior muscles, and NMJ staining on the extensor digitorum longus muscle of PGC1α-mTg female mice (Supplementary Fig. 3). The gene expression obtained in several types of muscles was similar to that obtained in Fig. 2. The mean AChR area of PGC1α-mTg female mice was significantly larger (Supplementary Fig. 3e,f).

Amyotrophic lateral sclerosis, duchenne muscular dystrophy are known to cause NMJ degeneration. Overexpression of PGC1α in skeletal muscle (transgenic mice) has been reported to improve muscle function in a mouse model of amyotrophic lateral sclerosis^21^, and duchenne muscular dystrophy^19,20^. In addition, overexpression of Dok-7 by adeno-associated virus has been reported to suppress NMJ degeneration and improve motor function in a mouse model of amyotrophic lateral sclerosis^8^. Another study revealed that Dok-7 overexpression by adeno-associated virus improved reinnervation and AChR cluster density, decreased fragmentation, and promoted NMJ regeneration after nerve injury^22^. We have shown that PGC1α regulates Dok-7 expression. Therefore, the above improvement in neuromuscular diseases by increased PGC1α expression may be mediated by increased Dok-7 expression.

### Exercise increased PGC1α and Dok-7 expression in skeletal muscle

**Figure 3:**
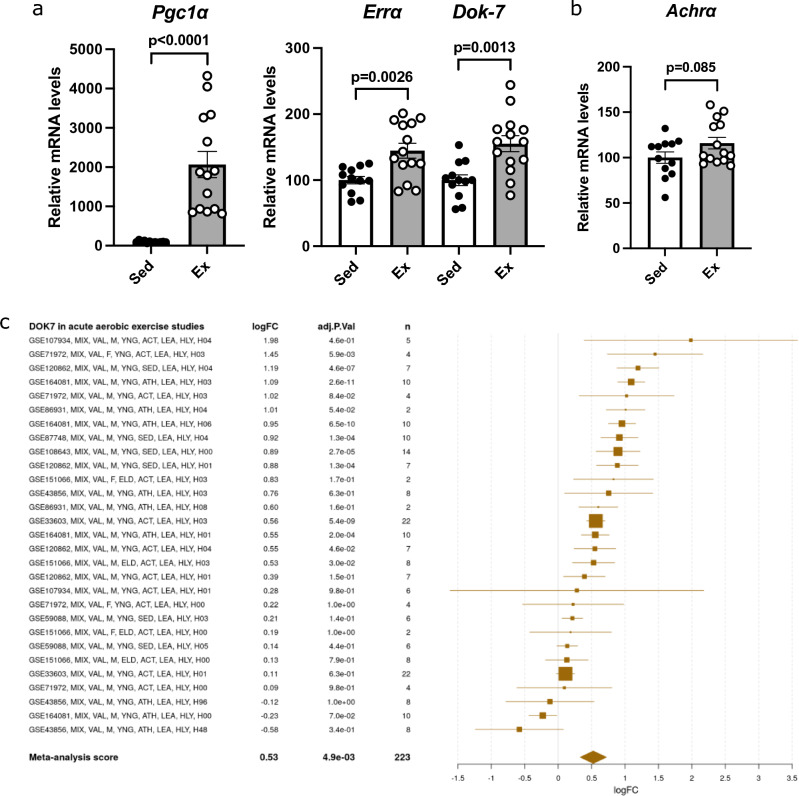

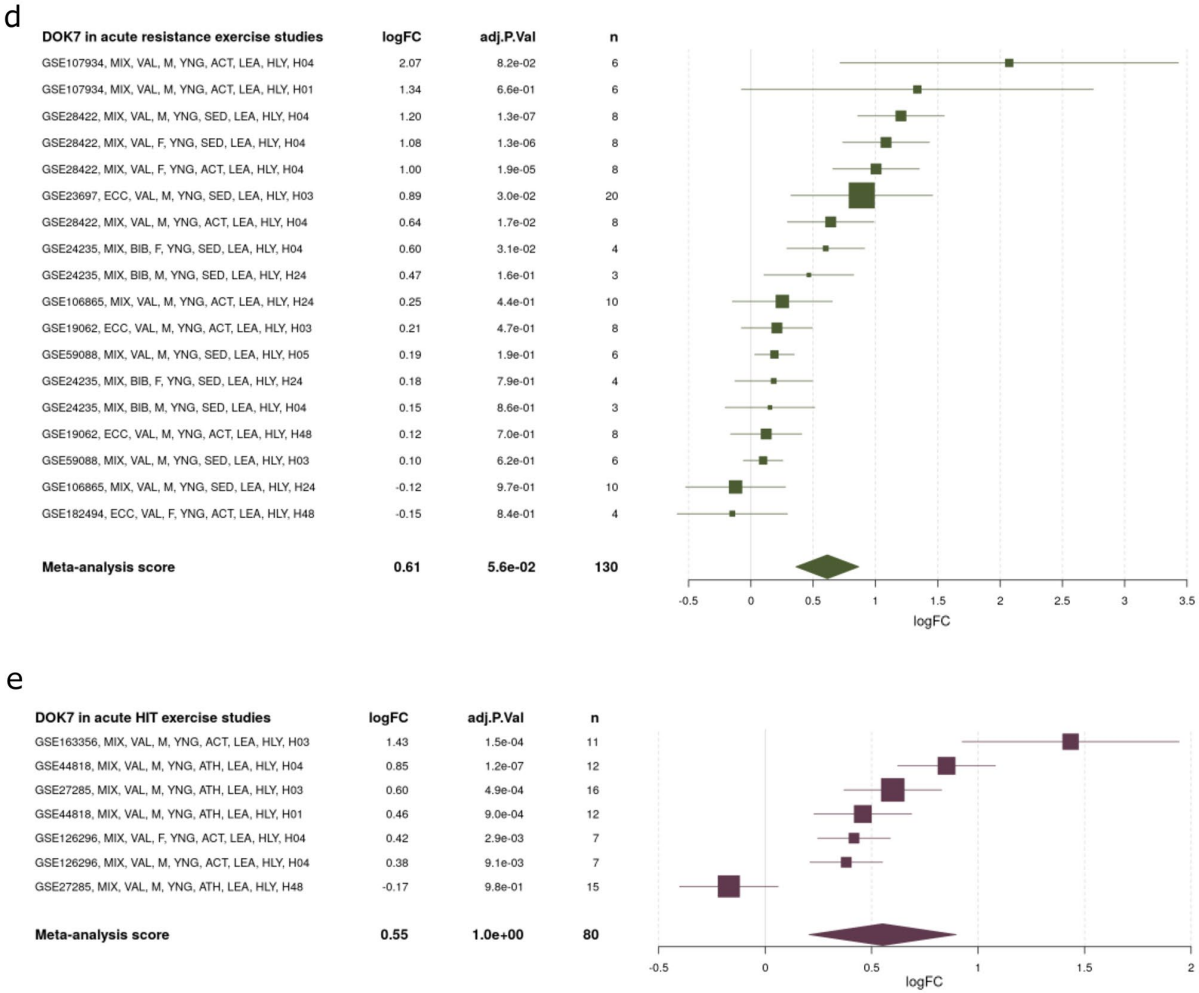
Increased peroxisome proliferator-activated receptor γ coactivator 1α (PGC1α) and downstream of tyrosine kinase-7 (Dok-7) expression in the skeletal muscle of after exercise. (**a**, **b**) Gene expression in the gastrocnemius muscle of 9-week-old male mice and female mice was analyzed using quantitative real-time PCR (N = 12 or 14). Data were normalized to 36B4 expression and expressed relative to sedentary mice (sed). The coefficients of variation are shown in Supplementary Table 2. The values are shown as the mean ± SE. (**c**–**e**) The online tool MetaMEx was used to investigate the expression of Dok-7 through various exercises. The forest plot obtained from this analysis was displayed.

Gene expression was analyzed in the gastrocnemius muscle of trained WT mice. The expression of PGC1α, ERRα and Dok-7 was increased in exercised mice (Fig. 3a). However, the same 6 h of exercise did not result in significant changes in AChR gene expression (Fig. 3b). In mice, continuous treadmill exercise at 28 m/min, 60 min/d, 5 d/week for 12 weeks has been reported to restore NMJ formation^23,24^. Furthermore, aging is known to cause NMJ fragmentation, but exercise attenuates age-related changes in NMJ^25^.

In addition, we examined the exercise-induced changes in the expression of Dok-7 using the online database of skeletal muscle transcriptomic response to exercise and inactivity (MetaMEx). This meta-analysis (MetaMEx) showed that PGC1α increased after acute aerobic exercise and after acute resistance exercise^26^. The meta-analysis included data from 11 studies of acute aerobic exercise, 8 studies of acute resistance exercise, 4 studies of acute high intensity interval training (HIT) exercise. The human meta-analysis confirmed that Dok-7 expression increased after acute aerobic exercise 1.38-fold (logFC 0.53), acute resistance exercise 1.47-fold (logFC 0.61), and HIT exercise 1.41-fold (logFC 0.55) (Fig. 3c–e).

Therefore, our results suggest that exercise-induced recovery of NMJ formation involves increased PGC1α and Dok-7 expression.

### PGC1α regulates Dok-7 gene expression in vitro

**Figure 4:**
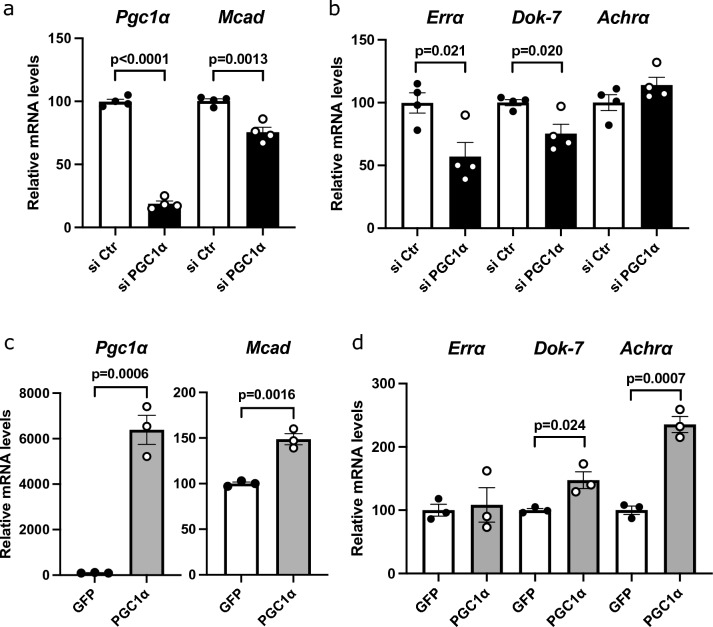
Downstream of tyrosine kinase-7 (Dok-7) gene expression regulated by peroxisome proliferator-activated receptor γ coactivator 1α (PGC1α) in vitro. (**a**, **b**) PGC1α was knocked down in muscle satellite cells. (**c**, **d**) PGC1α was overexpressed in muscle satellite cells. Gene expression was analyzed in gastrocnemius muscle-derived satellite cells using quantitative real-time PCR (**a**, **b**: N = 4; **c**, **d**: N = 3). Data were normalized to 36B4 expression and expressed relative to si control (si Ctr) or GFP. The values are shown as the mean ± SE.

Primary cultured cells (muscle satellite cells) were isolated from the skeletal muscle and used for in vitro experiments. In myotubes derived from muscle satellite cells, knockdown of PGC1α resulted in reduced expression of medium chain acyl CoA dehydrogenase (MCAD)—a target gene of PGC1α (Fig. 4a). The expression of ERRα and Dok-7 was reduced in these myotubes (Fig. 4b). AChRα gene expression was unchanged (Fig. 4b). Overexpression of PGC1α in myotubes derived from muscle satellite cells increased MCAD expression (Fig. 4c). ERRα expression was unchanged, whereas Dok-7 and AChRα expression were increased (Fig. 4d). These results were consistent with the in vivo results (Figs. 1a,b and 2a,b).

### PGC1α and ERRα work together to regulate Dok-7 expression

**Figure 5:**
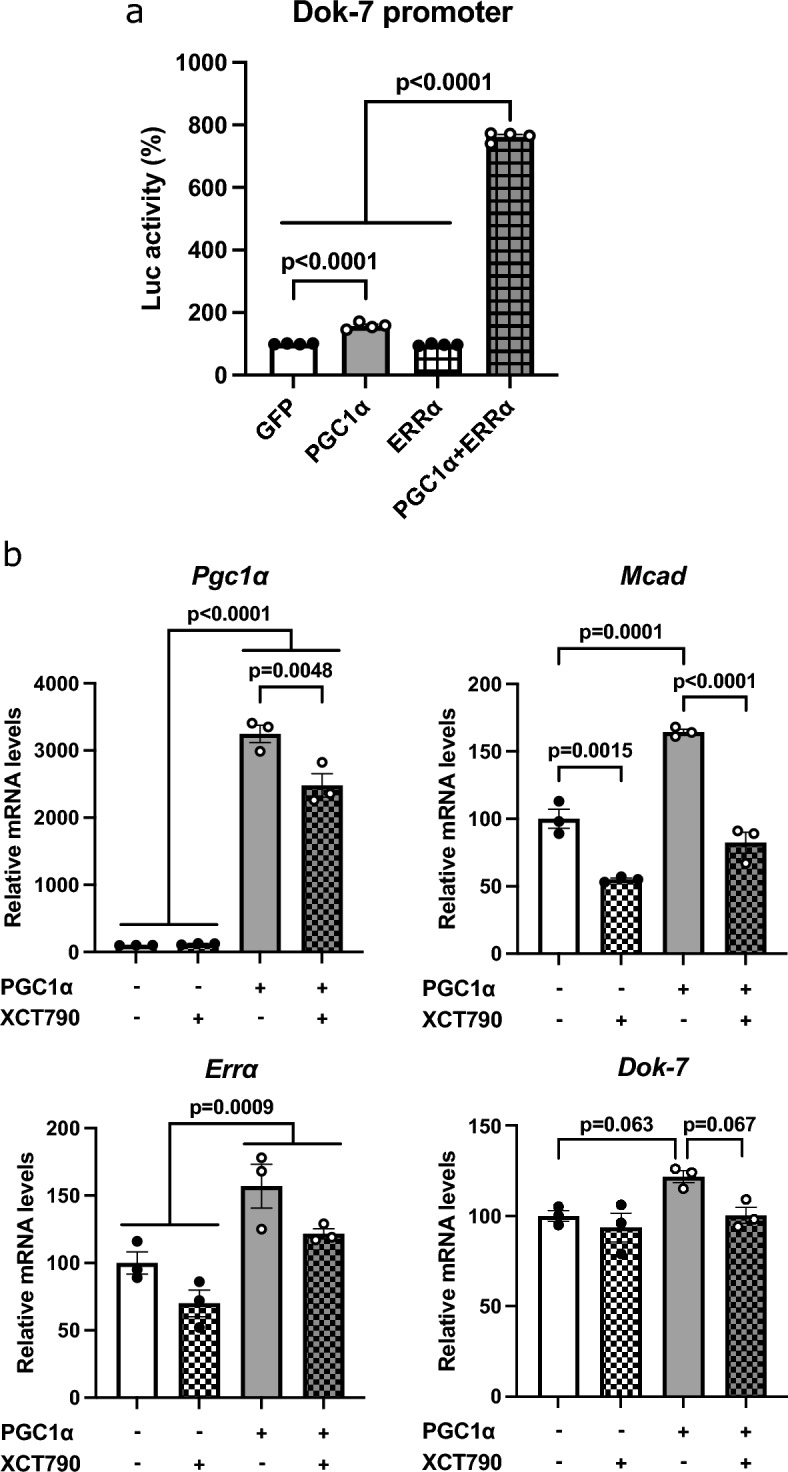
Downstream of tyrosine kinase-7 (Dok-7) expression is influenced by peroxisome proliferator-activated receptor γ coactivator 1α (PGC1α) and estrogen receptor-related receptor α (ERRα). The effect of increasing PGC1α and ERRα expression was examined by cotransfecting HEK293T cells with a reporter plasmid. (**a**) The constructs include a 2000 bp genomic promoter region and the first exon of the Dok-7 gene (− 2000 bp to + 73 bp, from the transcription start site) and the luciferase reporter gene (N = 4). The values are represented as the mean ± SE. (**b**) XCT790 was added to the PGC1α- overexpressing muscle satellite cells. Gene expression in gastrocnemius muscle-derived satellite cells was analyzed using quantitative real-time PCR (N = 3). Data were normalized to 36B4 expression and expressed relative to PGC1α (−) and XCT790 (−). The values are shown as the mean ± SE.

Because PGC1α increased Dok-7 expression, we hypothesized that Dok-7 is a new transcriptional target of PGC1α. We constructed a plasmid containing a sequence 2000 bp upstream of the Dok-7 transcription start site (− 2000 bp to + 73 bp) and the luciferase gene. In HEK293 cells, Dok-7 promoter activity was greatly increased upon cotransfection of PGC1α and ERRα, compared with transfection of either ERRα or PGC1α alone (Fig. 5a). The results indicate that PGC1α and ERRα work together to increase Dok-7 promoter activity. A truncated Dok-7 promoter (− 1047 bp) was used to measure luciferase activity. Promoter truncation reduced Dok-7 promoter activity upon coexpression of PGC1α and ERRα (Supplementary Fig. 4a). The Dok-7 promoter contains an ERR response element (ERRE) between  − 2000 bp and  − 1047 bp. Mutations in ERRE^27^ reduced Dok-7 promoter activity (Supplementary Fig. 4b). Further, Dok-7 promoter activity was measured by inserting mutations in ERR binding-sequences; however, decrease in promoter activity was not comparable with that of the truncated promoter. Thus, ERRE between  − 2000 bp and  − 1047 bp in the Dok-7 promoter is partly responsible for the increase in Dok-7 promoter activity by PGC1α and ERRα.

Finally, we investigated the effect of changes in ERRα activity on Dok-7 expression. The addition of XCT790, an inhibitor of ERRα activity, to myotubes derived from muscle satellite cells overexpressing PGC1α resulted in reduced expression of PGC1α and MCAD. (Fig. 5b). A decreasing trend in Dok-7 gene expression was observed with inhibitors of ERRα activity (Fig. 5b). These results suggest that PGC1α and ERRα activity induce in the expression of Dok-7 combined with the promoter activity results.

## Conclusion

**Figure 6:**
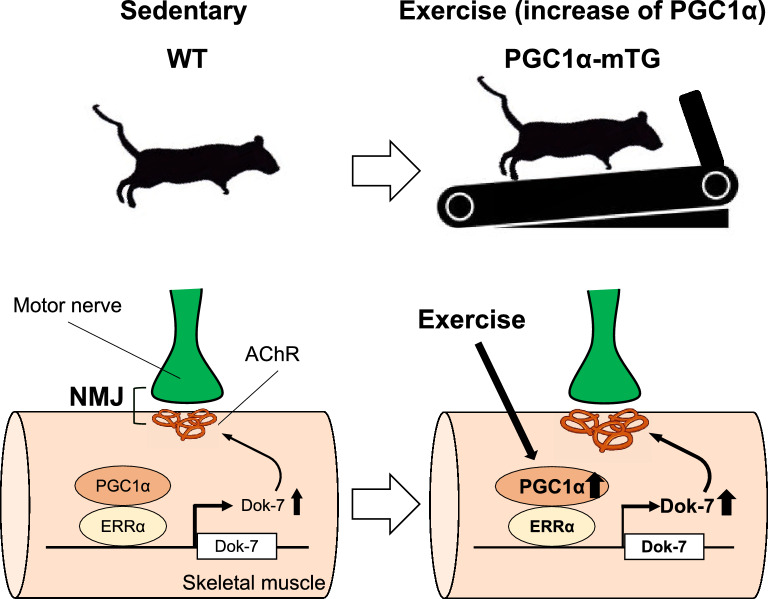
Gene expression of downstream of tyrosine kinase-7 (Dok-7) is regulated by peroxisome proliferator-activated receptor γ coactivator 1α (PGC1α) and estrogen receptor-related receptor α (ERRα). Regulation of increased Dok-7 expression and neuromuscular junction (NMJ) formation by PGC1α and ERRα. PGC1α is increased by exercise and functions as a transcriptional coactivator of nuclear receptors. PGC1α and ERRα work together to increase Dok-7 expression.

In this study, we showed that Dok-7 expression, which is important for NMJ formation, is regulated by PGC1α. PGC1α and ERRα work together to increase Dok-7 promoter activity. These results suggest that PGC1α is involved in the exercise-mediated restoration of NMJ formation (Fig. 6). Enhanced NMJ formation by PGC1α and ERRα is expected to improve muscle function and extend a healthy lifespan.

## Methods

### Animals

To control PGC1α ablation, we generated a conditional knockout version of the PGC1α gene using the Cre–loxP recombination system^28^. The genotypes of offspring were PGC1α flox/flox with Cre (PGC1α-mKO mice) and PGC1α flox/flox without Cre (WT mice). The experiment was performed on 8-week-old PGC1α-mKO male mice and 22- to 29-month-old female mice. PGC1α-mTg mice were generated as described^29^. In brief, the human α-skeletal actin promoter was used to express PGC1α in the skeletal muscle (C57BL/6 background). The experiment was performed on 10–12-week-old PGC1α-mTg male mice and 8-week-old female mice. The 9-week-old male C57BL/6J mice and 9-week-old female mice were exercised as described^30^. Mice were acclimated to moderate treadmill running (10 m/min for 15 min, shock intensity: 0.5 mA) for 1 week before the start of the experiment. After acclimation, mice performed 10% uphill treadmill running exercise at 15 m/min for 45 min periods 8 times (totally 6 h), with 5 min rest intervals. Exercised mice were euthanized immediately after exercise. Mice were maintained in a 12-h light/dark cycle at 24°C. Mice were cared for in accordance with the National Institutes of Health Guide for the Care and Use of Laboratory Animals and our institutional guidelines. All animal experiments were conducted with the approval of the Institutional Animal Care and Use Committee of Kyoto Prefectural University (No. KPU260407, review board: Dr. Yasuhiro Tsukamoto). The animals were euthanized by cervical dislocation and all efforts were made to minimize suffering. All animal experiments reported in this study were done in accordance with ARRIVE guidelines.

### Quantitative real-time RT-PCR analysis

Total RNA was prepared using TRIzol. cDNA was synthesized from 500 ng of total RNA using ReverTra Ace qPCR RT Master Mix with gDNA Remover. Gene expression was quantified with ABI PRISM 7000 using Thunderbird Next SYBR qPCR Mix, designed to detect cDNAs. Data were analyzed using the ΔΔCt method. All data obtained were normalized to 36B4 expression. The primers used are shown in Supplementary Table 1.

### Whole-mount staining of NMJs

For whole-mount staining, extensor digitorum longus muscles were fixed in 1% paraformaldehyde in PBS; permeabilized with 1% Triton X-100 in PBS; and incubated with anti-neurofilament-L and anti-synapsin-1 rabbit antibodies (1:500 and 1:1000), followed by Alexa 647-conjugated anti-rabbit IgG (1:2000) and Alexa 555-conjugated α-bungarotoxin (1:2000). The size (area) of AChR clusters was quantified using BZ-X800 Analyzer software.

### MetaMEx online tool

The online tool MetaMEx (www.metamex.eu) has already been developed^26^. For Dok-7 gene, the application displays an output for the different types of exercise protocols. Meta-analysis (MetaMEx) was performed on all sexes, all ages, lean weight, and healthy individuals. The bottom line of each graph shows the meta-analysis score.

### Muscle satellite cells

Gastrocnemius muscle-derived satellite cells were obtained as described^31^ using 8-week-old C57BL/6 J mice. The cells were cultured in growth medium (Dulbecco’s modified Eagle’s medium [DMEM] glucose (−) supplemented with 30% fetal bovine serum, 1% chicken–embryo extract, 1% GlutaMAX, 1% penicillin–streptomycin, and 10 ng/mL basic fibroblast growth factor) using 150-mm dishes coated with Matrigel.

### Cell culture in knockdown/overexpression of PGC1α in muscle satellite cells

Muscle satellite cells were plated at a density of 5 × 10^4^ cells per well in a 12-well plate containing growth medium. Muscle satellite cells were incubated in differentiation medium (DMEM containing 4.5 g/L glucose, supplemented with 2% horse serum) for 3 d, and then transfected with siRNA of PGC1α cultured in differentiation medium for 48 h.

pMX-derived expression plasmids containing PGC1α cDNA were expressed in muscle satellite cells as described^32^. Total RNA was isolated from the cells and analyzed by quantitative real-time RT-PCR.

### Plasmid constructs

pGL3-Dok-7 reporter plasmids included genomic promoter regions of the mouse Dok-7 gene (− 2000 bp to + 73 bp,  − 1047 bp to + 73 bp from the transcription start site) and the luciferase reporter gene.

### Transfection and luciferase assays

HEK293T cells were plated at a density of 8 × 10^4^ cells per well in a 24-well plate in DMEM containing 4.5 g/L glucose, supplemented with 10% fetal bovine serum, and transfected with the luciferase reporter plasmid (0.4 μg), expression plasmid (pCMX-PGC1α: 0.1 μg, pCMX-ERRα: 0.1 μg, pCMX-PGC1α + pCMX-ERRα: 0.1 μg + 0.1 μg), and pCMX-GFP (0.3 μg or 0.2 μg). phRL-TK vector (12.5 ng) was used as an internal control of transfection efficiency. The cells were lysed and assayed for luciferase activity using the Dual-Glo Luciferase Assay kit 24 h after transfection. Activity was calculated as ratio of firefly luciferase activity to Renilla luciferase activity (internal control).

### Treatment of muscle satellite cells with an ERRα inhibitor

Muscle satellite cells were plated at a density of 5 × 10^4^ cells per well in a 12-well plate in growth medium, incubated in differentiation medium for 3 d, and treated with XCT790 (20 μM) for 24 h. Total RNA was isolated from the cells and analyzed by quantitative real-time RT-PCR.

### Statistical analyses

Statistical comparison was performed by Student’s two-tailed unpaired *t*-test or one-way analysis of variance, followed by Tukey’s post hoc test for more groups. Data were checked for normality and equal variances between groups. *P* < 0.05 was considered statistically significant.

### Supplementary Information

Supplementary Information.

## Data Availability

The datasets generated and/or analyzed during the current study are available from the corresponding author upon reasonable request.
