# Supplementary material for: Peroxisome proliferator-activated receptor γ coactivator 1α regulates downstream of tyrosine kinase-7 (Dok-7) expression important for neuromuscular junction formation
Source: Sci Rep. 2024 Jan 20;14:1780. doi: 10.1038/s41598-024-52198-x (PMC10799880; doi:10.1038/s41598-024-52198-x)
Supplement: Supplementary file 1 — Supplementary Information. [file 41598_2024_52198_MOESM1_ESM.pdf]

Supplementary Materials

Peroxisome proliferator-activated receptor γ coactivator 1α regulates downstream of tyrosine kinase-7 (Dok-7) expression important for neuromuscular junction formation

Takumi Sugimoto[1, #)], *Chihiro Sakamaki[1, #)], Tokushi Kimura[1)], Takahiro Eguchi[2)], Shinji Miura[3)], Yasutomi Kamei[1, *)]

[#] co-first author: Takumi Sugimoto and Chihiro Sakamaki
[*]Corresponding author: Yasutomi Kamei, Ph.D.
Email: kamei@kpu.ac.jp

1) Laboratory of Molecular Nutrition, Graduate School of Environmental and Life Science, Kyoto Prefectural University, Kyoto, Japan.
2) Brain-Skeletal Muscle Connection in Aging Project Team, Geroscience Research Center, National Center for Geriatrics and Gerontology, Aichi, Japan.
3) Laboratory of Nutritional Biochemistry, Graduate School of Nutritional and Environmental Sciences, University of Shizuoka, Shizuoka, Japan.

Supplementary Figures S1-S4
Supplementary Tables S1-S2

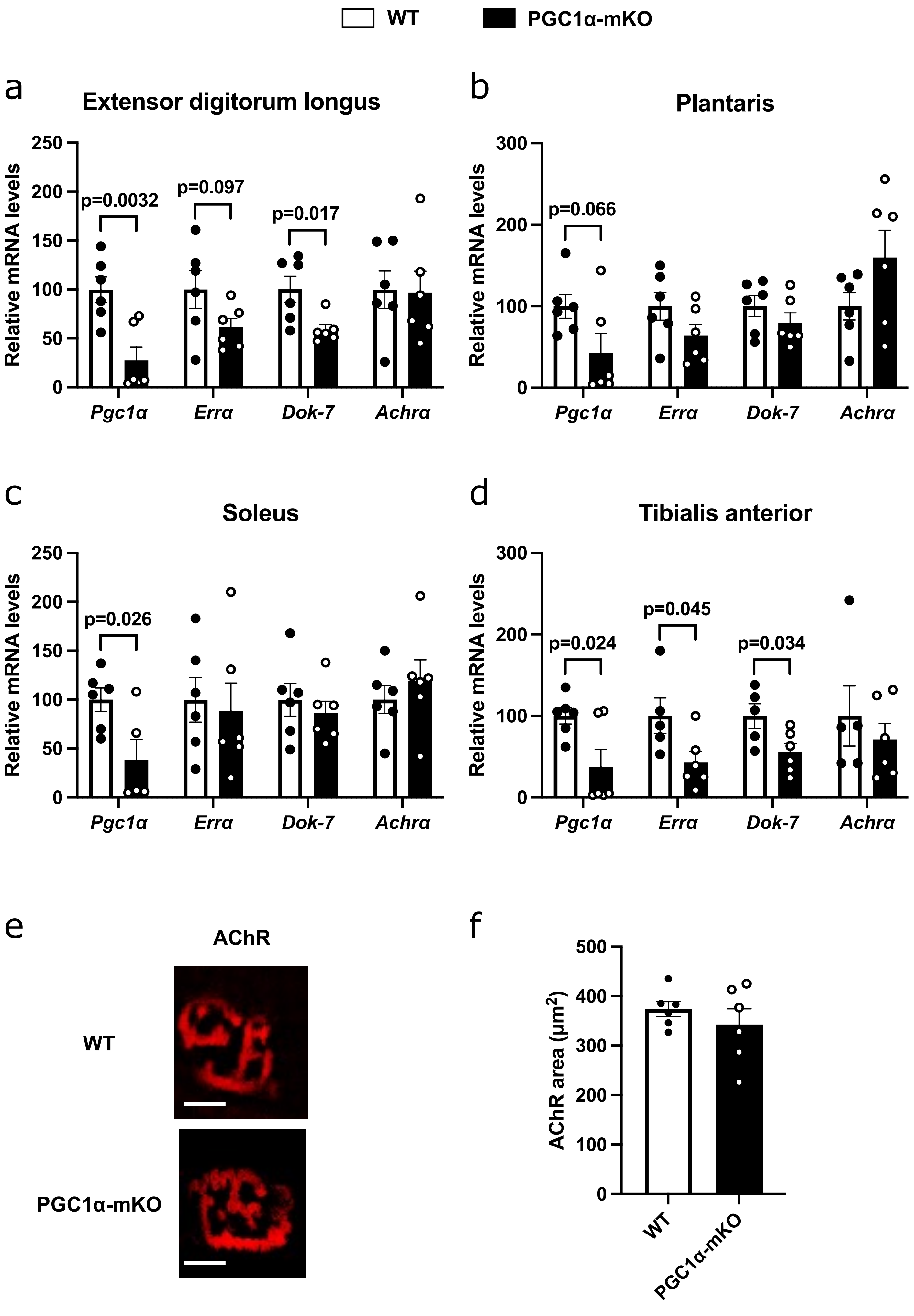
WT
PGC1α-mKO
a
Extensor digitorum longus
Relative mRNA levels
p=0.0032
p=0.097
p=0.017
Pgc1α
Errα
Dok-7
Achrα
b
Plantaris
p=0.066
c
Soleus
p=0.026
d
Tibialis anterior
p=0.024
p=0.045
p=0.034
e
AChR
WT
PGC1α-mKO
f
AChR area (μm²)

Supplementary Figure 1. Downstream of tyrosine kinase-7 (Dok-7) expression and neuromuscular junction (NMJ) formation are also changed in skeletal muscle-specific peroxisome proliferator-activated receptor γ coactivator 1α (PGC1α) knockout (PGC1α-mKO) female mice. (a-d) Gene expression in the extensor digitorum longus, plantaris, soleus and tibialis anterior muscle of 22- to 29-month-old PGC1α-mKO female mice was analyzed by quantitative real-time PCR (N = 6). Data were normalized to 36B4 expression and expressed relative to wild-type (WT) mice. (e) AChR staining of the extensor digitorum longus muscle. Representative images are shown for each group (N = 6). Scale bar = 15 μm. (f) The area of AChR clusters was quantified (N = 6).

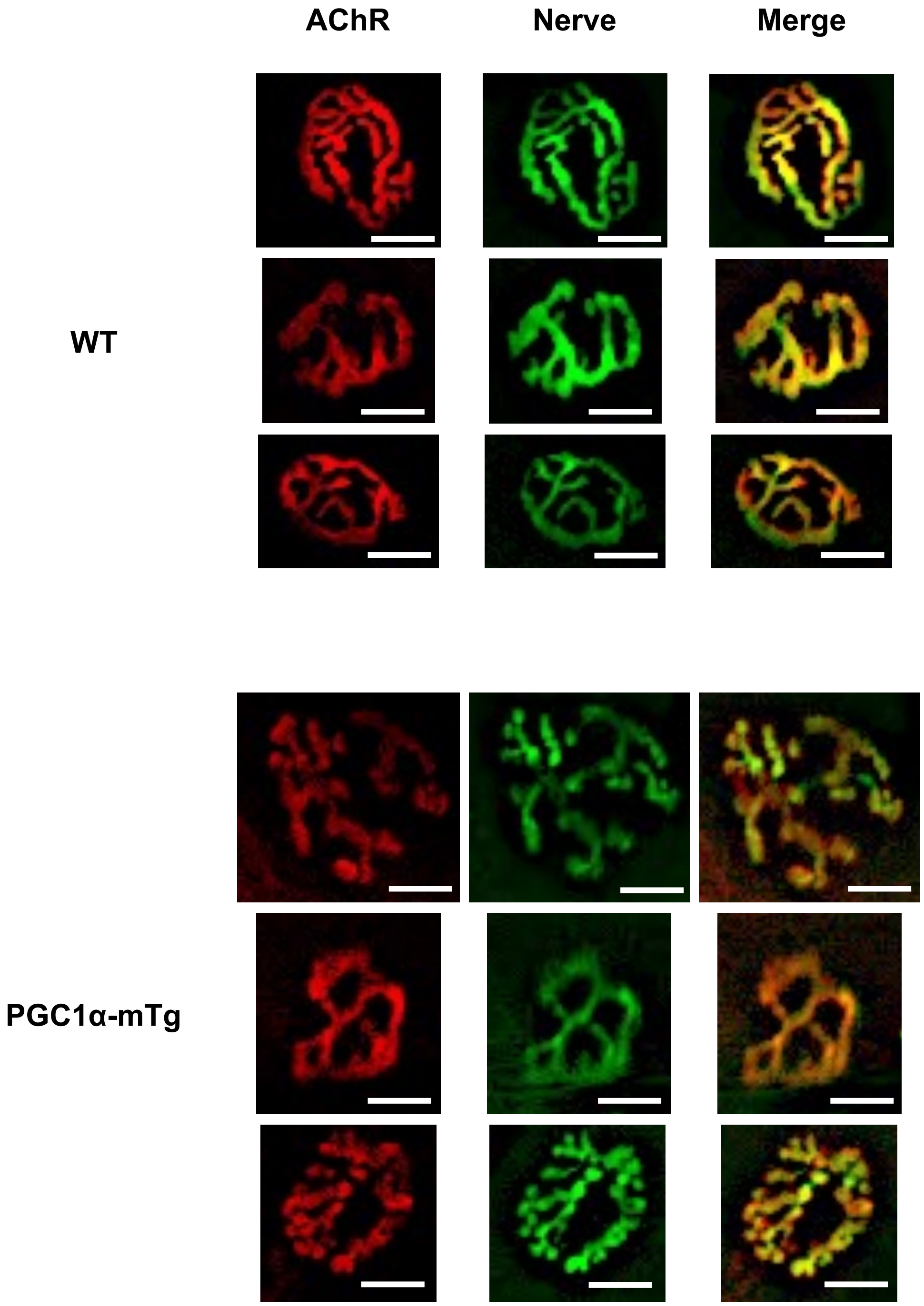

Supplementary Figure 2. Neuromuscular junction (NMJ) formation is normal in skeletal muscle-specific peroxisome proliferator-activated receptor γ coactivator 1α (PGC1α) overexpression (PGC1α-mTg) mice. AChR and nerve staining in the extensor digitorum longus muscle of 10-12-week-old PGC1α-mTg male mice. Scale bar = 15 μm.

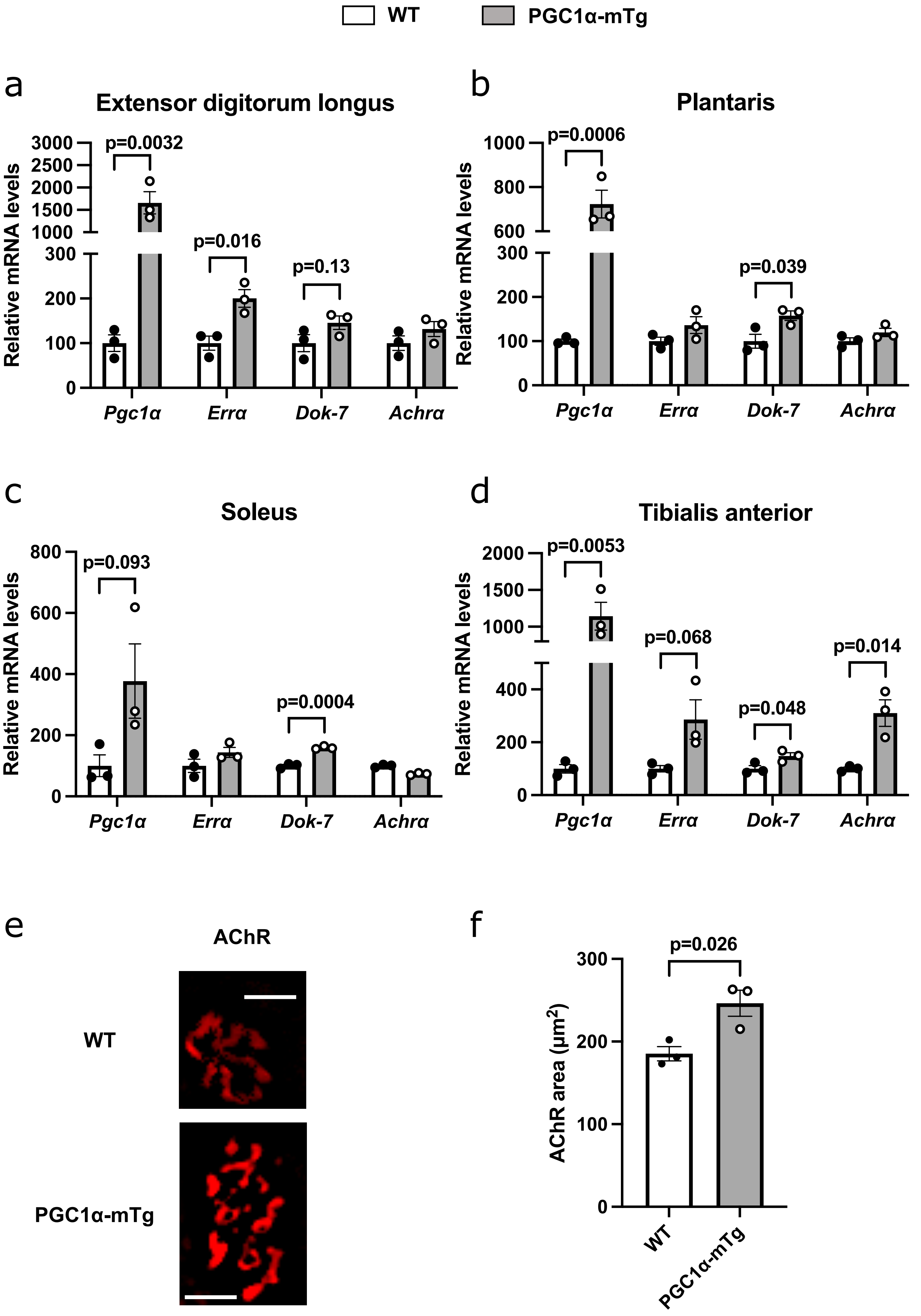

WT
PGC1α-mTg
a
Extensor digitorum longus
b
Plantaris
c
Soleus
d
Tibialis anterior
Relative mRNA levels
p=0.0032
p=0.016
p=0.13
p=0.0006
p=0.039
p=0.093
p=0.0004
p=0.0053
p=0.068
p=0.048
p=0.014
Pgc1α
Errα
Dok-7
Achrα
e
AChR
WT
PGC1α-mTg
f
p=0.026
AChR area (μm²)
WT
PGC1α-mTg

Supplementary Figure 3. Downstream of tyrosine kinase-7 (Dok-7) expression and neuromuscular junction (NMJ) formation are also changed in skeletal muscle-specific peroxisome proliferator-activated receptor γ coactivator 1α (PGC1α) overexpression (PGC1α-mTg) female mice. (a-d) Gene expression in the extensor digitorum longus, plantaris, soleus and tibialis anterior muscle of 8-week-old PGC1α-mTg female mice was analyzed by quantitative real-time PCR (N = 3). Data were normalized to 36B4 expression and expressed relative to wild-type (WT) mice. (e) AChR staining of the extensor digitorum longus muscle. Representative images are shown for each group (N = 3). Scale bar = 15 μm. (f) The area of AChR clusters was quantified (N = 3).

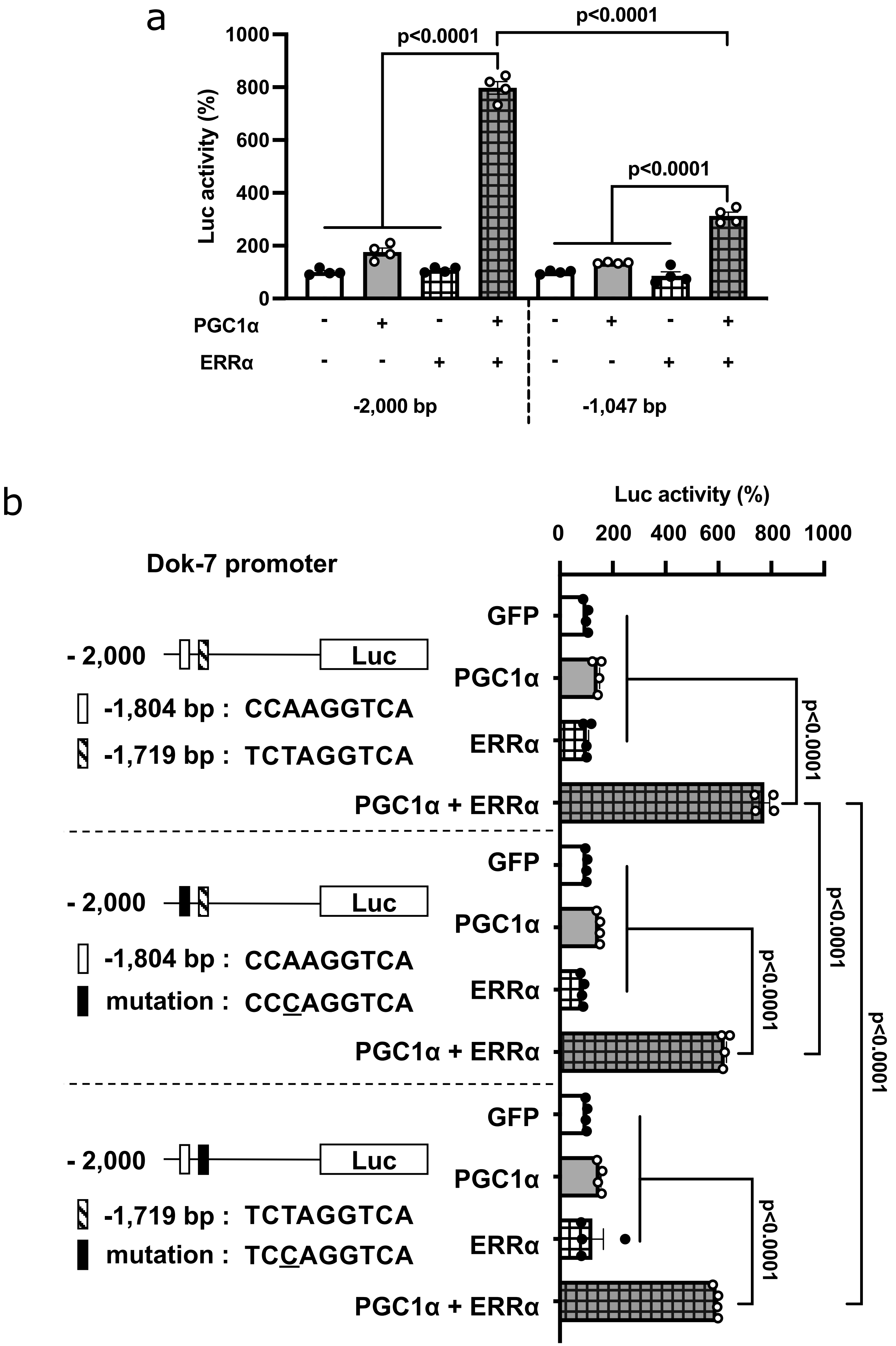

a
Luc activity (%)
1000
800
600
400
200
0
p<0.0001
p<0.0001
p<0.0001
PGC1α
ERRα
- + - +
- - + +
-2,000 bp
-1,047 bp
b
Luc activity (%)
0 200 400 600 800 1000
Dok-7 promoter
- 2,000
Luc
-1,804 bp : CCAAGGTCA
-1,719 bp : TCTAGGTCA
mutation : CCCAGGTCA
mutation : TCCAGGTCA
GFP
PGC1α
ERRα
PGC1α + ERRα
p<0.0001

Supplementary Figure 4. Promoter activity with mutations in the downstream of tyrosine kinase-7 (Dok-7) promoter. The effect of increasing peroxisome proliferator-activated receptor γ coactivator 1α (PGC1α) and estrogen receptor-related receptor α (ERRα) expression was examined by cotransfecting HEK293T cells with a reporter plasmid. (a) The constructs include a 2,000 bp and 1,074 bp genomic promoter region and the first exon of the Dok-7 gene (−2,000 bp to +73 bp and −1,074 bp to +73 bp, from the transcription start site) and the luciferase reporter gene ($N = 4$). The values are represented as the mean ± SE. (b) Measurement of Dok-7 promoter activity with mutations in ERR response element (ERRE) ($N = 4$). The values are represented as the mean ± SE.

Supplementary Table 1. Primer sequences used in quantitative real-time PCR. Primer sequences used for quantitative real-time PCR are listed in the Table.

Gene name	Primer Sequence (5'→3')
Pgc1α	F: CGGAAATCATATCCAACCAG
	R: TGAGGACCGCTAGCAAGTTTG
Errα	F: GCAAAGCCTTCTTCAAGAGGAC
	R: GGAGGCCGGACAGCTGT
Dok-7	F: TGGCGGACTCAAGGTCATG
	R: CCTGGATGCGTCACAGTGAAT
Achrα	F: CGTCTGGTGGCAAAGCT
	R: CCGCTCTCCATGAAGTT
Mcad	F: GATCGCAATGGGTGCTTTTGATAGAA
	R: AGCTGATTGGCAATGTCTCCAGCAAA
36B4	F: GGCCCTGCACTCTCGCTTTC
	R: TGCCAGGACGCGCTTGT

Supplementary Table 2. Coefficient of variation in in vivo experiments. The coefficient of variation (CV) was calculated as the standard deviation (SD) divided by the mean.

Figure 1										
	Pgc1α		*Errα*		*Dok-7*		*Achrα*		AChR area	
	WT	mKO	WT	mKO	WT	mKO	WT	mKO	WT	mKO
Mean	100	13	100	45	100	53	100	89	323	278
SD	29	22	47	18	23	22	39	35	61	66
CV	0.29	1.68	0.47	0.39	0.23	0.41	0.39	0.39	0.19	0.24

Figure 2										
	Pgc1α		*Errα*		*Dok-7*		*Achrα*		AChR area	
	WT	mTg	WT	mTg	WT	mTg	WT	mTg	WT	mTg
Mean	100	1311	100	242	100	138	100	187	275	342
SD	16	100	19	69	19	33	12	35	71	116
CV	0.16	0.08	0.19	0.29	0.19	0.24	0.12	0.19	0.26	0.34

Figure 3 a, b								
	Pgc1α		*Errα*		*Dok-7*		*Achrα*	
	Sed	Ex	Sed	Ex	Sed	Ex	Sed	Ex
Mean	100	2066	100	145	100	155	100	116
SD	23	1199	19	40	27	44	21	23
CV	0.23	0.58	0.19	0.28	0.27	0.28	0.21	0.20
